# Maternal caffeine intake during pregnancy is associated with risk of low birth weight: a systematic review and dose–response meta-analysis

**DOI:** 10.1186/s12916-014-0174-6

**Published:** 2014-09-19

**Authors:** Ling-Wei Chen, Yi Wu, Nithya Neelakantan, Mary Foong-Fong Chong, An Pan, Rob M van Dam

**Affiliations:** Saw Swee Hock School of Public Health, National University of Singapore and National University Health System, 16 Medical Drive, Block MD3, 117597 Singapore, Singapore; Yong Loo Lin School of Medicine, National University of Singapore and National University Health System, Singapore, Singapore; Clinical Nutrition Research Centre, Singapore Institute for Clinical Sciences, A*STAR, Singapore, Singapore; Department of Nutrition, Harvard School of Public Health, Boston, MA USA

**Keywords:** Coffee, Caffeine, Low birth weight, Small for gestational age, Intrauterine growth restriction, Systematic review, Meta-analysis

## Abstract

**Background:**

Considerable controversy exists regarding the relation between maternal caffeine intake during pregnancy and risk of low birth weight (birth weight <2,500 g). We aim to assess this association using a systematic review and dose–response meta-analysis of prospective studies.

**Methods:**

Potential articles were identified by searching MEDLINE and SCOPUS databases through 17 July 2013. Two authors independently extracted information on study design, participant characteristics and estimates of associations. Random-effects models were used to derive the summary relative risks (RRs) and corresponding 95% confidence intervals (CIs). Dose–response relationships were assessed using generalized least-squares trend estimation.

**Results:**

In our meta-analysis, we included 13 prospective studies: 9 with low birth weight as a binary outcome variable (90,747 participants and 6,303 cases) and 6 with birth weight as a continuous outcome variable (10,015 participants; 2 studies reported both types of outcomes). Compared with the reference category with no or very low caffeine intake, the RR (95% CI) of low birth weight was 1.13 (1.06 to 1.21; *I*^*2*^ 0.0%) for low intake (50 to 149 mg/day), 1.38 (1.18 to 1.62; *I*^*2*^ 31.9%) for moderate intake (150 to 349 mg/day), and 1.60 (1.24 to 2.08; *I*^*2*^ 65.8%) for high intake (≥350 mg/day). In the dose–response analysis, each 100-mg/day increment in maternal caffeine intake (around one cup of coffee) was associated with 13% (RR 1.13, 1.06 to 1.21) higher risk of low birth weight. The association persisted in strata defined according to various study characteristics. Moderate (−33 g, 95% CI −63 to −4; *I*^*2*^ 0.3%) and high (−69 g, 95% CI −102 to −35; *I*^*2*^ 0.0%) caffeine intakes were also associated with a significantly lower birth weight as compared with the reference category.

**Conclusions:**

Higher maternal caffeine intake during pregnancy was associated with a higher risk of delivering low birth weight infants. These findings support recommendations to restrict caffeine intake during pregnancy to low levels.

**Electronic supplementary material:**

The online version of this article (doi:10.1186/s12916-014-0174-6) contains supplementary material, which is available to authorized users.

## Background

Globally, it has been estimated that 15.5% of all infants are born low birth weight, defined as birth weight less than 2,500 g [1]. Low birth weight is not only associated with neonatal mortality and morbidity [1,2], but also with a higher risk of chronic diseases such as type 2 diabetes and cardiovascular diseases in adult life [3].

Caffeine is a plant alkaloid found mainly in coffee, tea, cola soft drinks and cocoa [4]. It is the most commonly used psychoactive substance [5]. Upon ingestion, caffeine is rapidly absorbed and readily passes the placental barrier [6,7]. The main enzyme (cytochrome P450 1A2) involved in caffeine metabolism is absent in both the placenta and the fetus which can lead to caffeine accumulation in fetal tissues [6,8,9]. The half-life of caffeine doubles in the mother during pregnancy as the rate of caffeine metabolism decreases from the first to third trimester [10,11]. This delayed clearance of caffeine leads to higher exposure to caffeine for the fetus. Exposure to caffeine can also lead to vasoconstriction in the uteroplacental circulation, which may in turn affect fetal growth and development [12,13].

As many women consume caffeine-containing food and beverages during pregnancy, the possible harmful effects of caffeine intake on fetal and birth outcomes warrant evaluation [4,14,15]. A number of studies have examined the relationship of maternal caffeine intake with low birth weight with mixed results [15,16]. Therefore, we systematically reviewed the available prospective epidemiological studies and conducted a meta-analysis on the association of maternal caffeine intake during pregnancy with risk of low birth weight and related outcomes, such as small for gestational age (SGA) and intrauterine growth restriction (IUGR).

## Methods

This meta-analysis was conducted and reported in accordance with the Meta-analysis of Observational Studies in Epidemiology (MOOSE) guideline [see Additional file 1] [17].

### Search strategy

Two investigators (L-WC and YW) searched MEDLINE and SCOPUS databases through 17 July 2013 with no language restriction. SCOPUS is an abstract and citation database of peer-reviewed literature that includes all the contents from the EMBASE database [18]. The search was based on combinations of synonyms for caffeine (including its chemical name, coffee and tea) and birth weight (including low birth weight, SGA and IUGR). The detailed search strategy is shown in Additional file 2.

### Selection criteria

Studies were included if they met the following criteria: (1) the study reported data from an original, peer-reviewed study (that is, not review articles or meeting abstracts); (2) the study was a prospective cohort study or nested case–control study; and (3) the authors reported the risk estimates of low birth weight associated with maternal caffeine intake (estimated total caffeine intake or coffee intake as a proxy for total caffeine intake) during pregnancy. We excluded studies that only presented crude estimates or did not consider potential confounding by smoking and studies conducted in unhealthy populations (for example, type 1 diabetes or infertility). We also excluded animal studies, case reports or case series, cross-sectional studies, retrospective case–control studies and other studies that assessed caffeine intake after the occurrence of outcome.

Low birth weight was defined as birth weight less than 2,500 g and SGA was defined as birth weight less than the 10^th^ percentile for gestational age. IUGR is officially defined as estimated fetal weight less than the 10^th^ percentile for gestational age [19,20], but the definition used in the included studies was based on birth weight (thus similar to SGA). Birth weight difference was defined as the difference in birth weight in the exposed (caffeine consumers) and unexposed (non- or very light- caffeine consumers) groups.

The study selection was independently conducted by two authors (L-WC and YW). We also considered non-English articles with help from colleagues who are proficient in these languages. The inter-rater agreement was fairly good (Kappa statistic = 0.64; Kappa coefficients that range from 0.61 to 0.80 indicate ‘substantial agreement’ [21]). Discrepancies were resolved by discussion with a third investigator (RMvD).

### Data extraction

For included studies, information on study, participants, measurement of exposure and outcome, effect estimates and their standard errors (or related statistics) were extracted independently by two investigators (L-WC and YW) using a standardized extraction form. Discrepancies were resolved by discussion with a third investigator (RMvD). Study quality assessment was done by considering characteristics such as study design, number of cases and participants, method of exposure assessment and adjustment of confounders. Sengpiel *et al*. defined SGA using three methods and we abstracted the effect estimates for outcome defined using Skjaerven’s method as similar methods were used in other studies included in our meta-analysis [9].

### Statistical analysis

Multivariable-adjusted odds ratios (ORs), hazard ratios (HRs) or relative risks (RRs) have been used in different studies, and we chose RRs as measures of risk estimates in the meta-analysis because the incident rate was low and OR and HR thus approximated RR [22]. For studies that reported birth weight as a continuous variable, the multivariable-adjusted birth weight differences (exposed minus unexposed) were used. Low birth weight, SGA and IUGR were treated as a single outcome in our primary analysis as they are closely related, but we also conducted stratified analysis based on different outcomes. For studies that reported results for more than one outcome [23-25], results for low birth weight were used in the main analysis due to simplicity and uniformity of its definition.

Different studies used different cutoff points for the caffeine intake categories. To combine the risk estimates from different categories in different studies, we assigned the median value for each category of caffeine intake. When lower and upper boundaries were presented for the category, we assigned the midpoint as an estimate of the median caffeine intake. If the upper boundary of the highest category was not provided, we assumed that the boundary had the same amplitude as the second-highest category [26]. If the lower boundary of the lowest category was not provided, we assumed the lower boundary to be zero [26]. Two European studies only reported coffee consumption in cups but not total caffeine intake [27,28] and we estimated caffeine intake based on the commonly cited conversion method (107 mg caffeine per cup of coffee) [29]. Several studies reported results for more than one period of maternal caffeine exposure (Table 1). We used the results for average caffeine intake during the whole pregnancy period if available [6,23]. Subsequently, we used the results for the assessment period most frequently used (first to second trimesters) in studies with only one exposure period. Nonetheless, we also included results from the other assessment periods in stratified analyses where possible.

**Table 1:** **Characteristics of prospective studies on caffeine intake in relation to low birth weight**

**First author, year**	**Country**	**Study design**	**Number of cases**	**Total number**	**Age**	**Exposure**	**Method of exposure assessment**	**Period of exposure assessed**	**Outcome**	**Adjustments**
Martin, 1987 [30]	United States	Cohort	70	3,654	<30 y: 69%	Caffeine	Interviewer-administered questionnaire	Early pregnancy	Low birth weight	Gestational age, ethnicity, parity, smoking
					≥30 y: 31%					
Olsen, 1991 [27]	Denmark	Cohort	391	11,591	<30 y: 71%	Coffee	Self-administered questionnaire	First to second trimester	Low birth weight	Smoking, social group, parity, alcohol intake (did not adjust maternal age for this outcome but did for birth weight)
					≥30 y: 29%					
Mills, 1993 [23]	United States	Cohort	21	352	<30 y: 48%	Caffeine	Interview	First, third trimesters and over the whole pregnancy^a^	Low birth weight^a^, intrauterine growth restriction	Maternal age, income, education, pre-pregnancy weight, height, ethnicity, parity, smoking and alcohol intake
					≥30 y: 52%					
Spinillo, 1994 [28]	Italy	Nested case–control	347	1,041	Case mean: 27.4 y	Coffee	NA	Variable, asked at prenatal visit or at delivery	Intrauterine growth restriction	Smoking, maternal age, marital status, parity, pre-pregnancy weight, BMI, weight gain, previous low birth weight, fetal sex, 1^st^ trimester hemorrhage, hypertension, education, social class, alcohol intake
					Control mean: 29.3 y					
Grosso, 2001 [31]	United States	Cohort	189	2,714	<30 y: 38%	Caffeine	Interviewer-administered questionnaire	Before 16 weeks gestations	Intrauterine growth restriction	Smoking, height, antenatal weight gain, preeclampsia during index pregnancy, parity and bleeding during the third trimester.
					≥30 y: 62%					
Bracken, 2003 [24]	United States	Cohort	108	2,291	<30 y: 47%	Caffeine	Interview	First trimester^a^ & third trimester^b^	Low birth weight^a^, intrauterine growth restriction	Maternal age, parity, number of prior pregnancies, marital status, ethnicity, education, height, smoking during the third trimester and weight
					≥30 y: 53%					
CARE study group, 2008 [6]	United Kingdom	Cohort	343	2,635	Mean 30.0 y	Caffeine	Interviewer-administered questionnaire (validated)	First, second, third trimesters and over the whole pregnancy^a^	Intrauterine growth restriction	Maternal age, weight, height, ethnicity, parity, neonatal gestational age at delivery and sex, smoking and alcohol intake
Bakker, 2010 [25]	The Netherlands	Cohort	331	7,346	Mean 29.7 y	Caffeine	Self-administered questionnaire (postal)	Third trimester	Low birth weight^a^, Small for gestational age	Gestational age at visit, maternal age, education, ethnicity, parity, smoking, alcohol intake, height, BMI at intake, nutritional intake, folic acid supplement use, maternal pregnancy complications and fetal sex
Sengpiel, 2013 [9]	Norway	Cohort	4,503	59,123	<30 y: 46%	Caffeine	Self-administered FFQ (validated)	First to second trimester	Small for gestational age	Maternal age, pre-pregnancy BMI, parity, history of preterm delivery, fetal sex, nausea during second trimester, smoking, passive smoking, nicotine intake from other sources, alcohol intake, energy intake, maternal education, marital status and household income
					≥30 y: 54%					

^a^data used for the main analysis; ^b^data not used because they were collected postnatally (after the occurrence of outcome). BMI, body mass index; FFQ, food frequency questionnaire; NA, not available; y, year.

We first conducted analyses based on different levels of caffeine consumption. We identified four levels of caffeine consumption based on assigned median caffeine consumption level: (1) reference category, (2) low caffeine consumption (50 to 149 mg/day), (3) moderate caffeine consumption (150 to 349 mg/day) and (4) high caffeine consumption (≥350 mg/day). The median caffeine levels for the reference category were 0 mg/day in five studies [24,30-33] and up to 50 mg/day in another four studies [6,9,28,34]; three studies have higher median caffeine levels for the reference category due to broad categorization [25,27,35]. The median caffeine levels for the highest category were >700 mg/day in three studies [25,27,35] and we used the second highest category (350 to 700 mg/day) of these studies to represent ‘high caffeine consumption’ in order to improve comparability with other studies.

Effect estimates of the individual studies were combined using the random-effects method as described by DerSimonian and Laird [36], which considers both within-study and between-study variations. The Cochran Q test and *I*^*2*^ statistic were used to evaluate statistical heterogeneity among studies [37,38], and *I*^*2*^ values of 25%, 50% and 75% correspond to low, moderate and high degrees of heterogeneity, respectively [38].

We further conducted a dose–response analysis (for binary outcomes) using the generalized least-squares trend estimation (GLST) method as described by Greenland and Longnecker [39,40], which computes the trend from the correlated logRR estimates across caffeine consumption categories. We performed a two-stage GLST method that first estimates study-specific slopes before deriving an overall average slope [40], because this method allowed us to include effect estimates from studies that only reported results for caffeine intake as a continuous variable. We tested for a potential non-linear relationship between maternal caffeine intake and birth weight using a restricted cubic spline random-effects model with three knots; the *P*-value for non-linearity was obtained by testing the null hypothesis that the spline term is equal to 0.

We conducted stratified analyses and meta-regression analyses to assess potential sources of heterogeneity by different study-level characteristics. We conducted sensitivity analysis to assess the influence of each individual study by omitting one study at a time and calculating the summary RR for the remaining studies. Publication bias was evaluated with the Egger’s regression test, Begg’s adjusted rank correlation test and visual inspection of funnel plot [41,42]. All tests were performed using STATA version 11.2 (StataCorp, College Station, TX, USA), and two-sided *P*-values <0.05 were considered statistically significant.

## Results

**Figure 1:**
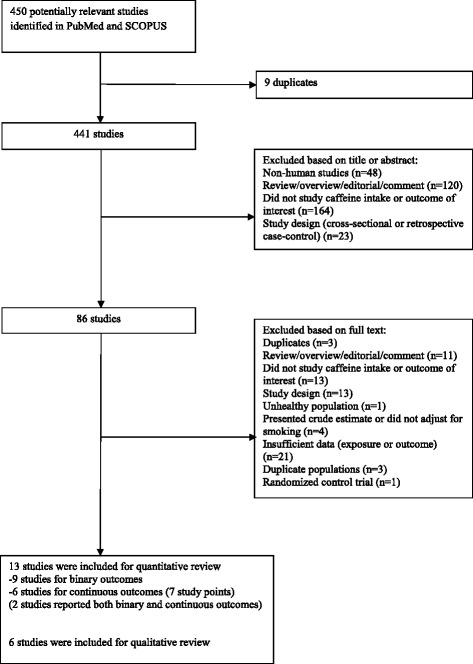
**Flow chart of study selection.**

The flow diagram with details of the study selection is shown in Figure 1. We included nine prospective studies [6,9,23-25,27,28,30,31] on caffeine intake and low birth weight, IUGR or SGA involving 90,747 participants and 6,303 cases (Table 1). In a secondary analysis, six prospective studies [6,30,32-35] on caffeine intake with birth weight difference as an outcome variable were included [see Additional file 3]. In total, thirteen studies were included for the quantitative review (two studies reported both binary and continuous birth weight outcomes [6,30]). An additional six studies that reported birth weight difference were only included for qualitative review because they did not report data for more than two categories of caffeine intake [9,24,27,43-45] [see Additional file 3]. All studies in this meta-analysis were conducted in the United States, Canada or Europe.

**Figure 2:**
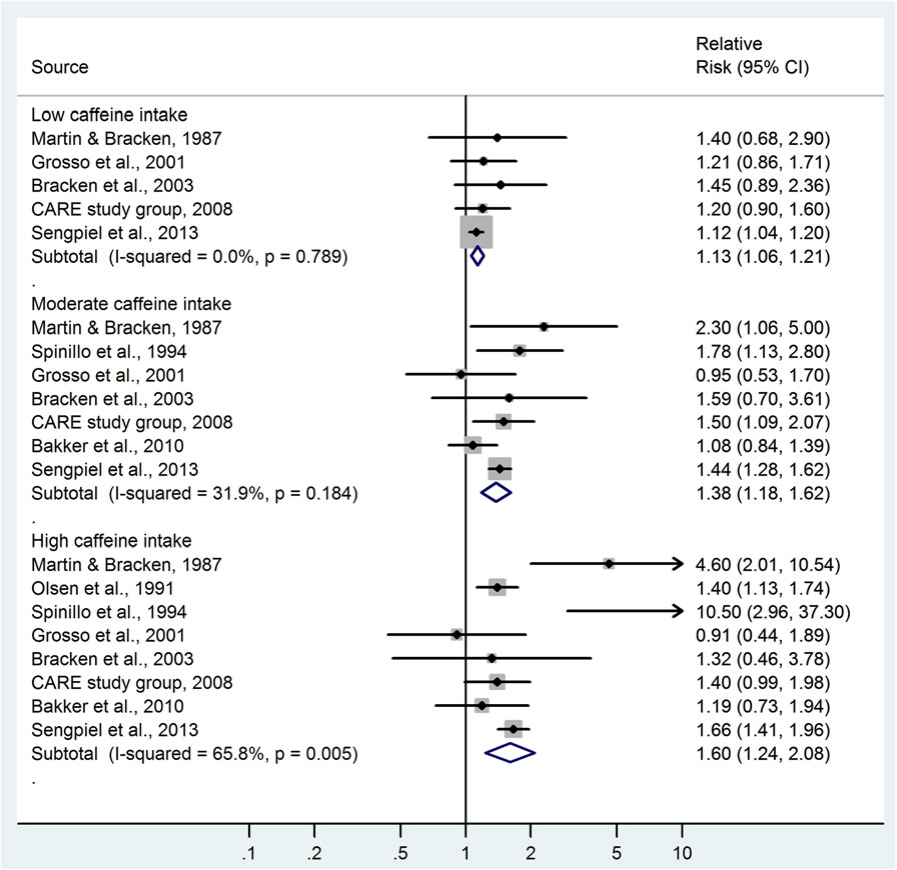
**Relative risks of low birth weight/IUGR/SGA according to maternal caffeine intake.** Low caffeine intake: 50 to 149 mg/day; moderate caffeine intake: 150 to 349 mg/day; high caffeine intake: ≥350 mg/day. Black dots indicate study-specific effect estimates, sizes of the grey squares correspond to the weights of the studies, horizontal lines indicate 95% CIs, and diamonds indicate the summary estimates with their corresponding 95% CIs. CI, confidence interval; IUGR, intrauterine growth restriction; SGA, small for gestational age.

Figure 2 shows the RRs for the association between maternal caffeine intake and low birth weight (including IUGR and SGA). The summary RR was 1.13 (95% CI 1.06 to 1.21) for low caffeine intake (50 to 149 mg/day), 1.38 (95% CI 1.18 to 1.62) for moderate caffeine intake (150 to 349 mg/day) and 1.60 (95% CI 1.24 to 2.08) for high caffeine intake (≥350 mg/day), as compared with the reference category with no or very low caffeine intake. The heterogeneity in study results was low to moderate: *I*^*2*^ = 0.0% for low caffeine intake, 31.9% for moderate caffeine intake and 65.8% for high caffeine intake.

**Figure 3:**
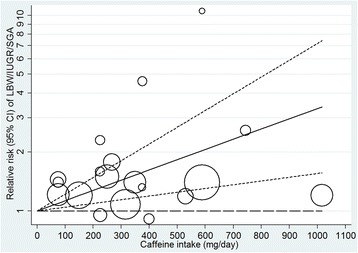
**Dose–response relationship between maternal caffeine intake and low birth weight/IUGR/SGA (n = 7).** Adjusted relative risks (RRs) and 95% CIs (dashed lines) are reported. Caffeine intake was modeled with a linear trend (*P*-value for non-linearity = 0.89) in a random-effects model. The vertical axis is on a log scale. The open circles represent the effect estimates from each study (the number of circles for a study depends on the number of caffeine intake categories in the study) and the size of the circles is proportional to the precision of the estimates. Mills *et al*.’s [23] and Sengpiel *et al*.’s [9] studies were not included in this graph as they did not provide sufficient results for categories of caffeine intake. CI, confidence interval; IUGR, intrauterine growth restriction; LBW, low birth weight; SGA, small for gestational age.

The dose–response relationship between maternal caffeine intake and low birth weight is shown in Figure 3. Because there was no evidence of departure from linearity (*P* = 0.89), we assumed a linear relationship. The summary RR was 1.13 (95% CI 1.06 to 1.21; Table 2) per 100-mg/day (equivalent to around one cup of coffee) and 1.45 (95% CI 1.20 to 1.76) per 300-mg/day increment of maternal caffeine intake. The overall heterogeneity was higher (*I*^*2*^ 82.4%) than for the categorical comparisons. However, the summary RRs did not substantially differ by various study characteristics including age of the mothers, region, year of publication, size of the study population, exposure assessment (total caffeine intake versus only coffee), questionnaire administration for exposure assessment (self-administered or interviewer-administered), assessed period of exposure during pregnancy, outcome definition (low birth weight, IUGR or SGA) and adjustment for smoking (a few or multiple categories) (Table 2). In a sensitivity analysis, the summary RRs ranged from 1.11 (95% CI 1.04 to 1.17) to 1.16 (95% CI 1.08 to 1.24) per 100-mg/day increment in maternal caffeine intake when we omitted one study at a time.

**Table 2:** **Stratified meta-analysis of caffeine intake (per 100-mg/day increment) and risk of low birth weight/IUGR/SGA**

**Characteristics**	**Number of studies**	**Summary RR (95% ** **CI)**	***P-*** **for-difference**	***P*** **-for-heterogeneity**	***I*** ^***2***^ **(95% ** **CI)**
**All studies**	9	1.13 (1.06 to 1.21)		<0.01	82.4 (65.4 to 89.1)
**Region**					
United States	4	1.20 (0.95 to 1.52)	Ref.	0.01	75.5 (32.3 to 91.1)
Europe	5	1.12 (1.05 to 1.19)	0.60	<0.01	87.2 (69.2 to 92.7)
**Year of publication**					
In or after 2000	5	1.10 (1.04 to1.16)	0.29^a^	0.07	53.9 (0.0 to 81.0)
Before 2000	4	1.27 (1.02 to 1.58)		<0.01	89.1 (74.8 to 95.3)
**Study population**					
≥2,500	6	1.11 (1.04 to1.18)	Ref.	<0.01	86.0 (68.9 to 91.8)
<2,500	3	1.28 (1.14 to 1.44)	0.25	0.45	0.0 (0.0 to 89.6)
**Study design**					
Cohort	8	1.11 (1.04 to 1.18)	Ref.	<0.01	81.0 (59.1 to 88.7)
Nested case–control	1	1.33 (1.16 to 1.52)	0.21	-	-
**Exposure**					
Caffeine	7	1.13 (1.06 to 1.21)	Ref.	0.01	67.4 (0.0 to 83.4)
Coffee	2	1.16 (0.91 to 1.48)	0.93	<0.01	91.9 (NA)
**Outcome** ^**b**^					
LBW	5	1.12 (1.02 to 1.23)	Ref.	<0.01	75.8 (40.6 to 90.1)
IUGR	5	1.12 (1.00 to 1.25)	0.78	0.04	59.5 (0.0 to 82.8)
SGA	2	1.13 (1.07 to 1.18)	0.85	0.14	53.9 (NA)
**Age** ^**c**^					
<30 years	4	1.17 (1.05 to 1.31)	Ref.	<0.01	88.6 (73.4 to 95.1)
≥30 years	5	1.13 (1.08 to 1.19)	0.54	0.32	15.1 (0.0 to 69.2)
**Method of exposure assessment**					
Interviewer-based	5	1.17 (1.01 to 1.36)	Ref.	0.01	68.2 (17.9 to 87.7)
Self-administered	3	1.08 (1.01 to 1.16)	0.42	<0.01	90.7 (70.5 to 95.2)
NA	1	1.33 (1.16 to 1.52)	0.38	-	-
**Exposure period assessed** ^**b**^					
First trimester	3	1.17 (0.90 to 1.53)	Ref.	<0.01	83.0 (48.0 to 94.4)
First to second trimesters	2	1.09 (0.99 to 1.21)	0.62	<0.01	95.3 (NA)
Whole pregnancy	2	1.13 (1.04 to 1.24)	0.99	0.40	0.0 (NA)
Third trimester	2	1.06 (1.00 to 1.12)	0.64	0.63	0.0 (NA)
NA	1	1.33 (1.16 to 1.52)	0.51	-	-
**Adjustment for smoking** ^**d**^					
Fine	7	1.12 (1.04 to 1.20)	Ref.	<0.01	82.0 (59.1 to 89.6)
Crude	2	1.24 (0.89 to 1.72)	0.52	<0.01	91.7 (NA)
**Median population caffeine intake**					
<200 mg/d	5	1.15 (1.05 to 1.26)	0.32^a^	0.02	66.7 (0.0 to 85.1)
≥200 mg/d	3	1.10 (1.01 to 1.21)		<0.01	83.9 (51.8 to 94.7)
NA	1	1.40 (0.84 to 2.32)		-	-

^a^ *P*-value was obtained by modeling year of publication and median of assigned doses as continuous variables; ^b^total number of study is more than 9 because some studies reported additional (usable) results for a different outcome or exposure period; ^c^mean age <30 years or ≥30 years. If mean age is not available, classification was based on whether the majority of the population (>50%) is <30 years or ≥30 years; ^d^fine adjustment for smoking refers to studies that adjusted for amount of smoking or studies that adjusted for smoking using a biomarker; crude adjustment refers to studies that did not adjust for amount of smoking. *I* ^*2*^, I-squared; IUGR, intrauterine growth restriction; LBW, low birth weight; NA, not available; Ref., reference. RR, relative risk; SGA, small for gestational age.

**Figure 4:**
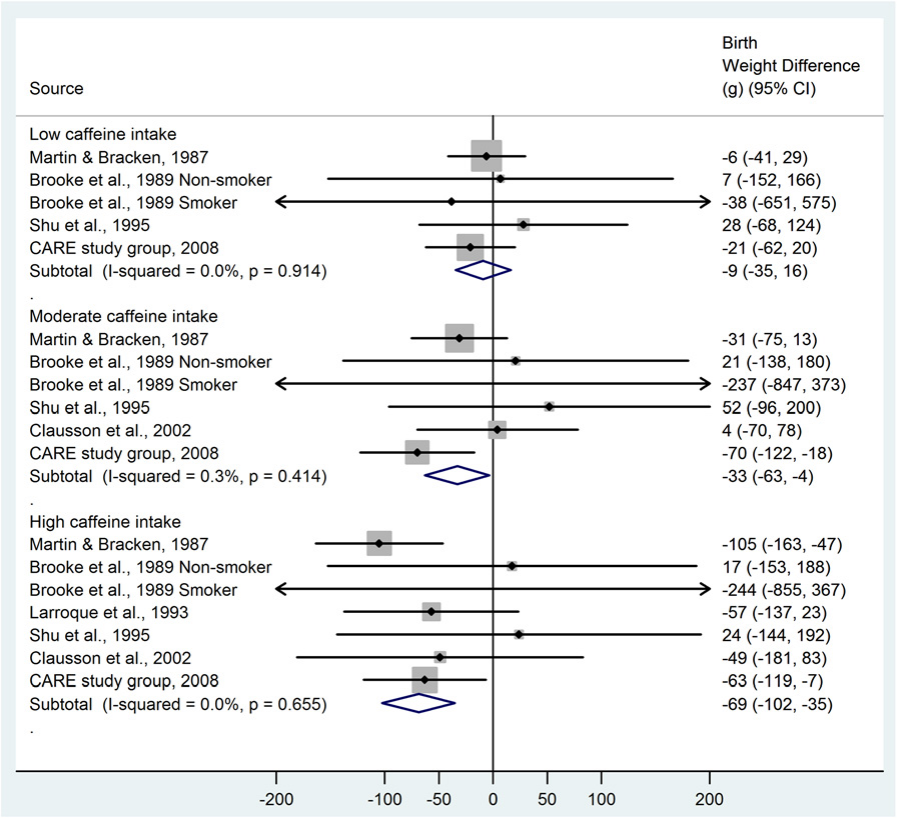
**Birth weight difference according to maternal caffeine intake.** Low caffeine intake: 50 to 149 mg/day; moderate caffeine intake: 150 to 349 mg/day; high caffeine intake: ≥350 mg/day. Black dots indicate study-specific effect estimates, sizes of the grey squares correspond to the weights of the studies, horizontal lines indicate 95% CIs and diamonds indicate the summary estimates with their corresponding 95% CIs. CI, confidence interval.

We also conducted a meta-analysis of studies of caffeine intake and birth weight as a continuous outcome variable. As compared with the reference group with no or very low caffeine intake, birth weight was 9 g lower in the low caffeine intake group (95% CI −16 to 35), 33 g lower in the moderate caffeine intake group (95% CI 4 to 63), and 69 g lower in the high caffeine intake group (95% CI 35 to 102) (Figure 4). The heterogeneity in study results was low (*I*^2^ = 0.0% for low and high caffeine intakes, and 0.3% for moderate caffeine intake). As the results in the study by Brooke *et al*. were reported separately for smokers and non-smokers, the potential impact of including two data points from the same study was assessed by excluding either of the data points, but the heterogeneity remained low (*I*^2^ = 0.0% for low and high caffeine intakes, and around 13% for moderate caffeine intake). For studies that were included for qualitative review only [see Additional file 3] [9,24,27,43-45], higher maternal caffeine intake was consistently associated with lower birth weight and this association was significant in five out of six studies [9,24,27,43,45].

There was no suggestion of publication bias based on Egger’s test (*P* = 0.22), Begg’s test (*P* = 0.60) or the funnel plot [see Additional file 4].

## Discussion

The findings from this meta-analysis of prospective studies suggest that maternal caffeine intake is associated with a higher risk of delivering an infant with low birth weight. Low caffeine intake (50 to 149 mg/day) was associated with a 13%, moderate caffeine intake (150 to 349 mg/day) with a 38%, and high caffeine intake (≥350 mg/day) with a 60% higher risk of low birth weight as compared with very low or no caffeine intake. These results suggest a graded relationship between caffeine intake and low birth weight. In a dose–response analysis, each 100-mg/day increment in maternal caffeine intake (about one cup of coffee) was associated with a 13% higher risk for low birth weight. The association persisted across strata defined by various study and participant characteristics.

Most previous reviews on maternal caffeine intake and low birth weight only qualitatively summarized the available evidence [15,16,46]. The most recent quantitative reviews only considered studies published up to year 1996 [47,48]. The meta-analysis conducted by Fernandes *et al*. suggested that pregnant women consuming more than 150 mg caffeine per day had a significantly higher risk of low birth weight compared with those with a lower caffeine intake [48]. This meta-analysis included only five studies and pooled crude effect estimates; confounding by factors such as smoking and maternal age is, thus, a concern. Similarly, in the other older meta-analysis, Santos *et al*. reported an association between high maternal caffeine intake and a significantly lower offspring’s birth weight and higher risks of having low birth weight and IUGR newborns [47]. However, the study only compared the extreme groups and the influence of moderate and low caffeine consumption during pregnancy remained unclear. Moreover, this meta-analysis combined estimates from cohort studies and case–control studies, which may be more prone to selection and recall biases.

To date, only one randomized controlled trial has studied the effect of a reduction in caffeine intake during pregnancy on birth weight [49]. This Danish study randomized 1,207 pregnant women into two groups that received either caffeinated instant coffee or decaffeinated instant coffee starting at about 20 weeks of gestation and, thus, only evaluated the effect of caffeine reduction in the second half of pregnancy. The estimated mean caffeine intake of the caffeinated coffee group was 182 mg/day higher than that of the decaffeinated coffee group. After adjusting for gestational age, pre-pregnancy BMI, parity and smoking at entry to the study, the mean birth weight for babies born to the caffeinated group was 16 g (95% CI −40 to 73) lower than that of the decaffeinated group. Although the difference was not significant, when statistical uncertainty is considered, it is compatible with our results of a 33 g lower birth weight (95% CI 4 to 63) for moderate caffeine intake (150 to 349 mg/day) as compared with no or very low caffeine intake.

Our meta-analysis has several strengths. We searched databases with no language restriction to increase the completeness of studies identification. In addition, we included only prospective studies and, thus, reduced the influence of selection bias, recall bias and reverse causation in our results. Lastly, we not only compared the highest category with the lowest categories but divided maternal caffeine intake into four levels and also conducted a dose–response analysis.

However, our study also has several limitations that have to be considered in the interpretation of our findings. Measurement error in the assessment of caffeine intake may have affected the results. The included studies relied on questionnaires for caffeine assessment. However, in most studies an interviewer-administered questionnaire was used, which may have improved the completeness of their data collection. Furthermore, validation studies have shown that self-reports of major sources of caffeine such as coffee and tea are, in general, reasonably accurate and reliable [50,51]. Another potential limitation was that two of the included studies (conducted in Italy and Denmark) used coffee consumption as the proxy of total caffeine intake. However, in these European populations coffee consumption is high and in a similar Swedish study coffee was the predominant source (accounting for 76%) of all caffeine ingested by pregnant women [52]. In our stratified analysis, the summary estimates did not differ substantially by assessment of coffee versus total caffeine intake. The caffeine assessments in the remaining studies were reasonably comprehensive. Most studies considered at least the intakes of coffee, tea and caffeinated-soft drinks [6,9,23,24,30-35,44], while many also additionally considered other sources such as cocoa products [6,9,23,30,31,33-35] and caffeine-containing medication [6,23,30,34]. Consumption of energy drinks containing a relatively high amount of caffeine has been becoming more common, especially since the mid-2000s [53,54]. However, most included studies in this meta-analysis were conducted before that period. Furthermore, the contribution of energy drinks to total caffeine intakes was found to be small in two recently published studies included in this meta-analysis—1% of caffeine intake in the CARE study and 7% (‘sugar sweetened caffeinated-soft drinks including energy drinks’) in Sengpiel *et al*.’s study [6,9] as well as other studies in pregnant women or women of child-bearing age [55,56]. Another issue is that three of the included studies had a relatively high median caffeine level for reference category [25,27,35], probably reflecting the high caffeine intake in these populations. However, this should have resulted in more conservative pooled effect estimates as it would reduce the contrast in caffeine intake between the compared groups.

We combined low birth weight, SGA and IUGR in our main analysis. Birth weight is influenced by both duration of gestation and rate of fetal growth. Therefore, low birth weight can result from preterm delivery, insufficient fetal growth or both, whereas SGA and IUGR only reflect fetal growth. Nonetheless, in a recent meta-analysis by Maslova *et al*. [4], the authors did not observe a substantial association between maternal caffeine intake during pregnancy and the risk of preterm birth. Hence, it seems likely that the association between maternal caffeine intake and the risk of low birth weight in our meta-analysis was due to lower fetal growth rather than preterm delivery. Furthermore, in our stratified analysis the pooled effect estimates for associations with caffeine intake were similar for studies with low birth weight, SGA and IUGR as an outcome.

Residual confounding by unmeasured or imperfectly measured covariates should also be considered as a potential limitation of our meta-analysis. A majority of the included studies adjusted for potential confounders rather comprehensively. We only included studies that considered potential confounding by smoking either by adjusting for it in multivariable models or reporting that such adjustment had minimal influence on the results. In addition, most studies adjusted for gestational age, fetal sex and maternal characteristics including age, weight and height, parity, alcohol intake and socio-economic status.

Smoking tends to correlate with caffeine intake [9,23] and is a known risk factor for low birth weight [57]. Smoking is, therefore, an important potential confounder of the association between caffeine intake and low birth weight. The seven included studies that reported results for potential interaction between caffeine intake and smoking in relation to low birth weight [9,23,27,28,30,31,35] either did not suggest differences in association between smokers and non-smokers [9,23,28,30,31] or suggested a stronger association in non-smokers [27,35]. It is reassuring that associations between caffeine intake and birth weight outcomes were observed in non-smokers, because residual confounding by intensity of smoking is not a concern in this group. However, in a study comparing self-reported smoking status and smoking status defined by biomarker (serum cotinine level), the non-disclosure rate of active smoking was higher among pregnant women (22.9%) than non-pregnant women (9.2%) [58]. Using self-reported maternal smoking status to adjust for confounding by smoking may, thus, lead to residual confounding. Nonetheless, in the CARE study, maternal caffeine intakes during all three trimesters of pregnancy were associated with a higher risk of fetal growth restriction even after adjustment for salivary cotinine.

Pregnancy symptoms including nausea, vomiting and aversions to smells and taste are more common in healthy pregnancies. Women with healthy pregnancies are more likely to decrease their caffeine consumption in response to pregnancy symptoms [15] and there is a suggestion that pregnancy symptoms can partly account for the relationship of caffeine intake and adverse pregnancy outcomes, such as low birth weight. Nonetheless, in a recent large cohort study by Sengpiel *et al*., the observation that higher maternal caffeine intake was associated with higher SGA risk was not affected by adjustment for nausea [9]. Furthermore, in the CARE study nausea and vomiting in pregnancy was not associated with fetal growth restriction and did not modify the association between maternal caffeine intake and fetal growth restriction [59].

Thus, the results of the original studies do not suggest that confounding explains the association between caffeine intake and low birth weight. Nevertheless, residual confounding cannot be completely ruled out in observational studies.

In addition to methodological limitations of the original studies, publication bias can affect the results of meta-analyses. In our meta-analysis, statistical tests for publication bias or the funnel plot did not suggest publication bias, but this type of bias can never be fully ruled out.

We assumed a linear relationship in our dose–response analysis as there was no evidence of departure from linearity. Also, our analysis based on categories of caffeine intake suggested a graded association between caffeine intake and the risk of low birth weight. We acknowledge that in the conduct of a meta-analysis, the use of reported data for categories of intake instead of individual level data may have reduced our power to detect a non-linear association. However, our finding that there appears to be no clear threshold level of intake below which caffeine does not affect birth weight is in concordance with two original studies that examined dose–response relationships using sextiles [9] or spline regression analysis [6]. Both of these studies suggested a monotonic association between caffeine intake and risk of low birth weight with risk being elevated even for low intakes as compared with caffeine abstinence.

The exact mechanism through which caffeine may impair fetal growth remains unsettled. One of the hypothesized mechanisms is that caffeine increases the release of catecholamines, which may lead to vasoconstriction in the uteroplacental circulation and fetal hypoxia and eventually affect fetal growth and development [12,13]. Indeed, a 25% reduction in intervillous placental blood flow after maternal ingestion of just 200 mg of caffeine has been documented [6,13]. Another hypothesis is that caffeine increases the cellular concentration of cyclic AMP by inhibiting phosphodiesterase, an enzyme responsible for the breakdown of cyclic AMP [9,60]. A built-up of cyclic AMP may influence cell division or lead to catecholamine-mediated vasoconstriction, thus affecting fetal growth [31,61].

## Conclusions

This systematic review and meta-analysis supports the hypothesis that higher maternal caffeine intake during pregnancy is associated with a higher risk of delivering low birth weight infants. In our meta-analysis based on categories of caffeine consumption, the risk for low birth weight increased with increasing levels of caffeine intake. The risk for low birth weight was significantly higher even in the low (50 to 149 mg/day) and moderate (150 to 349 mg/day) caffeine intake groups, as compared with the reference group with no or very low caffeine intake. Our dose–response analysis also suggested a linear association with low birth weight across the range of caffeine intakes. This seems relevant for public health recommendations as it indicates that the risk of low birth weight may be elevated even for caffeine intakes below the recommended maximum limit of the current guidelines for pregnant women (300 mg/day by the World Health Organization and 200 mg/day by the Nordic Nutrition Recommendations and the American College of Obstetricians and Gynecologists [9,62]). Nonetheless, although our meta-analysis summarizes the best evidence available to date, we cannot exclude the possibility that potential biases, such as reverse causation and residual confounding by smoking or pregnancy symptoms, affected the observed association between caffeine intake and risk of low birth weight. Results from previous studies have highlighted the adverse impact of smoking and alcohol use during pregnancy on birth weight and other birth outcomes [63-65]. Although abstaining from smoking and alcohol consumption should be the mainstay of recommendations to lower risk of low birth weight, reducing caffeine intake during pregnancy may represent an additional strategy to optimize fetal growth. In conclusion, while a confirmation in large randomized controlled trials would be desirable, our results do provide further support for recommendations to limit caffeine intake during pregnancy to low levels.

## Additional files

Additional file 1:
**MOOSE Checklist.**
Additional file 2:
**Search terms for MEDLINE and SCOPUS.**
Additional file 3:
**Characteristics of prospective studies on caffeine intake and birth weight difference (n = 12).**
Additional file 4:
**Funnel plot for low birth weight/IUGR/SGA.**

## Abbreviations

BMI: body mass index
CI: confidence interval
FFQ: food frequency questionnaire
GLST: generalized least-squares trend estimation
HR: hazard ratio
IUGR: intrauterine growth restriction
LBW: low birth weight
MOOSE: meta-analysis of observational studies in epidemiology
OR: odds ratios
RR: relative risks
SGA: small for gestational age
