# Supplementary material for: Maternal caffeine intake during pregnancy is associated with risk of low birth weight: a systematic review and dose–response meta-analysis
Source: BMC Med. 2014 Sep 19;12:174. doi: 10.1186/s12916-014-0174-6 (PMC4198801; doi:10.1186/s12916-014-0174-6)
Supplement: Additional file 1: — MOOSE Checklist. [file 12916_2014_174_MOESM1_ESM.docx]

**Additional file 1. MOOSE Checklist.**

From: [Donna F. Stroup](http://jama.ama-assn.org/search?author1=Donna+F.+Stroup&sortspec=date&submit=Submit), PhD, MSc; [Jesse A. Berlin](http://jama.ama-assn.org/search?author1=Jesse+A.+Berlin&sortspec=date&submit=Submit), ScD; [Sally C. Morton](http://jama.ama-assn.org/search?author1=Sally+C.+Morton&sortspec=date&submit=Submit), PhD; [Ingram Olkin](http://jama.ama-assn.org/search?author1=Ingram+Olkin&sortspec=date&submit=Submit), PhD; [G. David Williamson](http://jama.ama-assn.org/search?author1=G.+David+Williamson&sortspec=date&submit=Submit), PhD; [Drummond Rennie](http://jama.ama-assn.org/search?author1=Drummond+Rennie&sortspec=date&submit=Submit), MD; [David Moher](http://jama.ama-assn.org/search?author1=David+Moher&sortspec=date&submit=Submit), MSc; [Betsy J. Becker](http://jama.ama-assn.org/search?author1=Betsy+J.+Becker&sortspec=date&submit=Submit), PhD; [Theresa Ann Sipe](http://jama.ama-assn.org/search?author1=Theresa+Ann+Sipe&sortspec=date&submit=Submit), PhD; [Stephen B. Thacker](http://jama.ama-assn.org/search?author1=Stephen+B.+Thacker&sortspec=date&submit=Submit), MD, MSc; for the Meta-analysis Of Observational Studies in Epidemiology (MOOSE) Group. Meta-analysis of Observational Studies in Epidemiology. A Proposal for Reporting JAMA. 2000;283(15):2008-2012. doi: 10.1001/jama.283.15.2008

|  | Reported on page | Comments |
| --- | --- | --- |
| **Reporting of background should include** | | |
| Problem definition | 5 | Background |
| Hypothesis statement | 5 | Background |
| Description of study outcomes | 5, 7 | Background, Methods |
| Type of exposure or intervention used | 5 | Background |
| Type of study designs used | 6 | Methods |
| Study population | 5 | Background |
| **Reporting of search strategy should include** | | |
| Qualifications of searchers (eg librarians and investigators) | 6 | Methods |
| Search strategy, including time period used in the synthesis and key words | 6 | Methods |
| Effort to include all available studies, including contact with authors | 6-7 | Methods |
| Databases and registries searched | 6 | Methods |
| Search software used, name and version, including special features used (eg explosion) | 6 | Methods |
| Use of hand searching (eg reference lists of obtained articles) | Did not use hand searching |  |
| List of citations located and those excluded, including justification | Figure | Figure 1 |
| Method of addressing articles published in languages other than English | 7 | Methods |
| Method of handling abstracts and unpublished studies | 6 | Methods |
| Description of any contact with authors | Did not contact author |  |
| **Reporting of methods should include** | | |
| Description of relevance or appropriateness of studies assembled for assessing the hypothesis to be tested | 6-7 | Methods |
| Rationale for the selection and coding of data (eg sound clinical principles or convenience) | 6-7 | Methods |
| Documentation of how data were classified and coded (eg multiple raters, blinding and interrater reliability) | 7 | Methods |
| Assessment of confounding (eg comparability of cases and controls in studies where appropriate) | 7 | Methods |
| Assessment of study quality, including blinding of quality assessors, stratification or regression on possible predictors of study results | 7, 10 | Methods |
| Assessment of heterogeneity | 9 | Methods |
| Description of statistical methods (eg complete description of fixed or random effects models, justification of whether the chosen models account for predictors of study results, dose-response models, or cumulative meta-analysis) in sufficient detail to be replicated | 7-10 | Methods |
| Provision of appropriate tables and graphics | Figure | Figure 1 |
| **Reporting of results should include** | | |
| Graphic summarizing individual study estimates and overall estimate | Figures and tables | Figure 2, Figure 3, Figure 4, Table 2 |
| Table giving descriptive information for each study included | Tables | Table 1, Additional file 3 |
| Results of sensitivity testing (eg subgroup analysis) | 11-12 | Results |
| Indication of statistical uncertainty of findings | Figures and tables | Figure 2, Figure 4, Table 2 |
| **Reporting of discussion should include** | | |
| Quantitative assessment of bias (eg publication bias) | 12 | Results, Additional file 4 |
| Justification for exclusion (eg exclusion of non-English language citations) | Figure | Figure 1 |
| Assessment of quality of included studies | 14-17 | Discussion |
| **Reporting of conclusions should include** | | |
| Consideration of alternative explanations for observed results | 14-18 | Discussion |
| Generalization of the conclusions (eg appropriate for the data presented and within the domain of the literature review) | 19-20 | Conclusions |
| Guidelines for future research | 19-20 | Conclusions |
| Disclosure of funding source | Acknowledgement | Acknowledgement |

Transcribed from the original paper within the Support Unit for Research Evidence (SURE), Cardiff University, United Kingdom. February 2011.
