# Supplementary material for: Maternal caffeine intake during pregnancy is associated with risk of low birth weight: a systematic review and dose–response meta-analysis
Source: BMC Med. 2014 Sep 19;12:174. doi: 10.1186/s12916-014-0174-6 (PMC4198801; doi:10.1186/s12916-014-0174-6)
Supplement: Additional file 2: — Search terms for MEDLINE and SCOPUS. [file 12916_2014_174_MOESM2_ESM.docx]

**Additional file 2. Search terms for MEDLINE and SCOPUS.**

**The following terms were used for the MEDLINE search:**

("caffeine"[MeSH Terms] OR "caffeine"[All Fields] OR "coffee"[MeSH Terms] OR "coffee"[All Fields] OR "tea"[MeSH Terms] OR "tea"[All Fields]) AND ("birth weight"[MeSH Terms] OR "birth weight"[All Fields] OR "infant, low birth weight"[MeSH Terms] OR "low birth weight"[All Fields] OR "infant, small for gestational age"[MeSH Terms] OR "small for gestational age"[All Fields] OR "SGA"[All Fields] OR "fetal growth retardation"[MeSH Terms] OR "fetal growth retardation"[All Fields] OR "fetal growth restriction"[All Fields] OR "intrauterine growth restriction"[All Fields] OR "IUGR"[All Fields])

**The following terms were used for the SCOPUS search:**

(“caffeine” OR “1,3,7-trimethlyxanthine” OR “coffee” OR “tea”) AND (“birth weight” OR “low birth weight” OR “small for gestational age” OR “SGA” OR “fetal growth restriction” OR “fetal growth retardation” OR “intrauterine growth restriction” OR “IUGR”)
