# Supplementary material for: Maternal caffeine intake during pregnancy is associated with risk of low birth weight: a systematic review and dose–response meta-analysis
Source: BMC Med. 2014 Sep 19;12:174. doi: 10.1186/s12916-014-0174-6 (PMC4198801; doi:10.1186/s12916-014-0174-6)
Supplement: Additional file 3: — Characteristics of prospective studies on caffeine intake and birth weight difference (n = 12). [file 12916_2014_174_MOESM3_ESM.docx]

| **Additional file 3. Characteristics of prospective studies on caffeine intake and birth weight difference (n=12).** | | | | | | | | | |
| --- | --- | --- | --- | --- | --- | --- | --- | --- | --- |
| **First author, year** | **Country** | **Total popu-lation** | **Study design** | **Age** | **Exposure** | **Method of exposure assessment** | **Period of exposure assessed** | **Outcome** | **Adjustments** |
| Kuzma, 1982^a^ [43] | United states | 5093 | Cohort | Mean 24.6 y | Caffeine | Self-administered questionnaire | NA | Birth weight difference | Gestational age, pre-pregnancy weight, weight gain, ethnicity, smoking, alcohol intake, parity, previous spontaneous abortions, frequency of illicit drug use |
| Fried, 1987^a^ [44] | Canada | 667 | Cohort | Mean 28.9 | Caffeine | Interview (24-h recall) | Over the pregnancy | Birth weight difference | Consumption of alcohol, cannabis, and nicotine, pre-pregnancy maternal weight, pregnancy weight gain, maternal height, pregnancy number, gestational age, sex of child, family income |
| Martin, 1987 [30] | United states | 3654 | Cohort | <30 y: 69% ≥30 y: 31% | Caffeine | Interview | Early pregnancy | Birth weight difference | Gestational age, parity, smoking, marital status, ethnicity |
| Brooke, 1989 [33] | United Kingdom | 1513 | Cohort | <30 y: 67%  ≥30 y: 33% | Caffeine | Interviewer-administered questionnaire | The week before interview | Birth weight difference (converted from birth weight ratio) | Gestational age, maternal height, infant sex, parity, and smoking through stratification |
| Olsen, 1991^a^ [27] | Denmark | 11591 | Cohort | <30 y: 71%  ≥30 y: 29% | Coffee | Self-administered questionnaire | First and second trimesters | Birth weight difference | Maternal age, smoking, parity, social group, tea consumption, and alcohol intake |
| Dar, 1992^a^ [45] | United States | 1341 | Cohort | Age range 18-35 y | Caffeine | Self-administered questionnaire | NA | Birth weight difference | Polychlorinated biphenyls exposure, gestational age, weight gain, usual weight, birth order, infant gender, smoking, clinic, alcohol intake during pregnancy and non-pregnancy, income, and urbanicity |
| Larro-que, 1993 [35] | France | 628 | Cohort | NA | Caffeine | Interview | First trimester | Birth weight difference | Smoking, alcohol intake, gestational age, infant sex, maternal age, parity, education, maternal height and weight |
| Shu, 1995 [32] | United states | 712 | Cohort | Mean age ranged from 26.1-27.9 y (stratified by smoking, alcohol drinking, and caffeine status) | Caffeine | Interview at around 13 weeks (telephone)*, self-administered questionnaire (postal) at 28 & 36 weeks | First*, second and third trimesters | Birth weight difference | Gestational age, parity, log of pre-pregnancy weight, smoking, income, bleeding during first trimester |
| Clau-sson, 2002 [34] | Sweden | 873 | Cohort | <30 y: 58% ≥30 y: 42% | Caffeine | Interview | Over the pregnancy | Birth weight difference | Maternal age, height, BMI, country of birth, parity, previous low birth weight, education, work, nausea, vomiting, fatigue, diabetes, hypertensive disorders & cotinine levels in the third trimester (smoking) |
| Bracken, 2003^a^ [24] | United states | 2291 | Cohort | <30 y: 47% ≥30 y: 53% | Caffeine | Interview | First trimester, third trimester^#^ | Birth weight difference | Smoking, gestational age, ethnicity, height, weight, gravidity, education, maternal age |
| CARE study group, 2008 [6] | United Kingdom | 2635 | Cohort | Mean 30.0 y | Caffeine | Interviewer-administered questionnaire (validated) | First, second, third trimesters and over the pregnancy* | Birth weight difference | Maternal age, weight, height, ethnicity, parity, neonatal gestational age at delivery and sex, smoking and alcohol intake |
| Seng-piel, 2013^a^ [9] | Norway | 59123 | Cohort | <30 y: 46%  ≥30 y: 54% | Caffeine | Self-administered FFQ (validated) | First to second trimester | Birth weight difference | Maternal age, pre-pregnancy BMI, parity, history of preterm delivery, fetal sex, nausea during second trimester, smoking, passive smoking, nicotine intake from other sources, alcohol intake, energy intake, maternal education, marital status, and household income |

Abbreviation: y, years; NA, not available; BMI, body mass index.

^a^Included for qualitative review only.

*Data used for this meta-analysis.

^#^Data not used because they were collected postnatally (after the occurrence of outcome).
