# Supplementary material for: Maternal caffeine intake during pregnancy is associated with risk of low birth weight: a systematic review and dose–response meta-analysis
Source: BMC Med. 2014 Sep 19;12:174. doi: 10.1186/s12916-014-0174-6 (PMC4198801; doi:10.1186/s12916-014-0174-6)
Supplement: Additional file 4: — Funnel plot for low birth weight/IUGR/SGA. [file 12916_2014_174_MOESM4_ESM.docx]

**Additional file 4. Funnel plot for low birth weight/IUGR/SGA.**

Abbreviation: IUGR, intrauterine growth restriction; SGA, small for gestational age; SE, standard error; RR, relative risk.

There was no suggestion of publication bias based on both Egger’s test (*p* = 0.22) and Begg’s test (*p* = 0.60).
